# Supplementary figures and images for: Innate Immune Response of TmToll-3 Following Systemic Microbial Infection in Tenebrio molitor
Source: Int J Mol Sci. 2023 Apr 4;24(7):6751. doi: 10.3390/ijms24076751 (PMC10095136; doi:10.3390/ijms24076751)

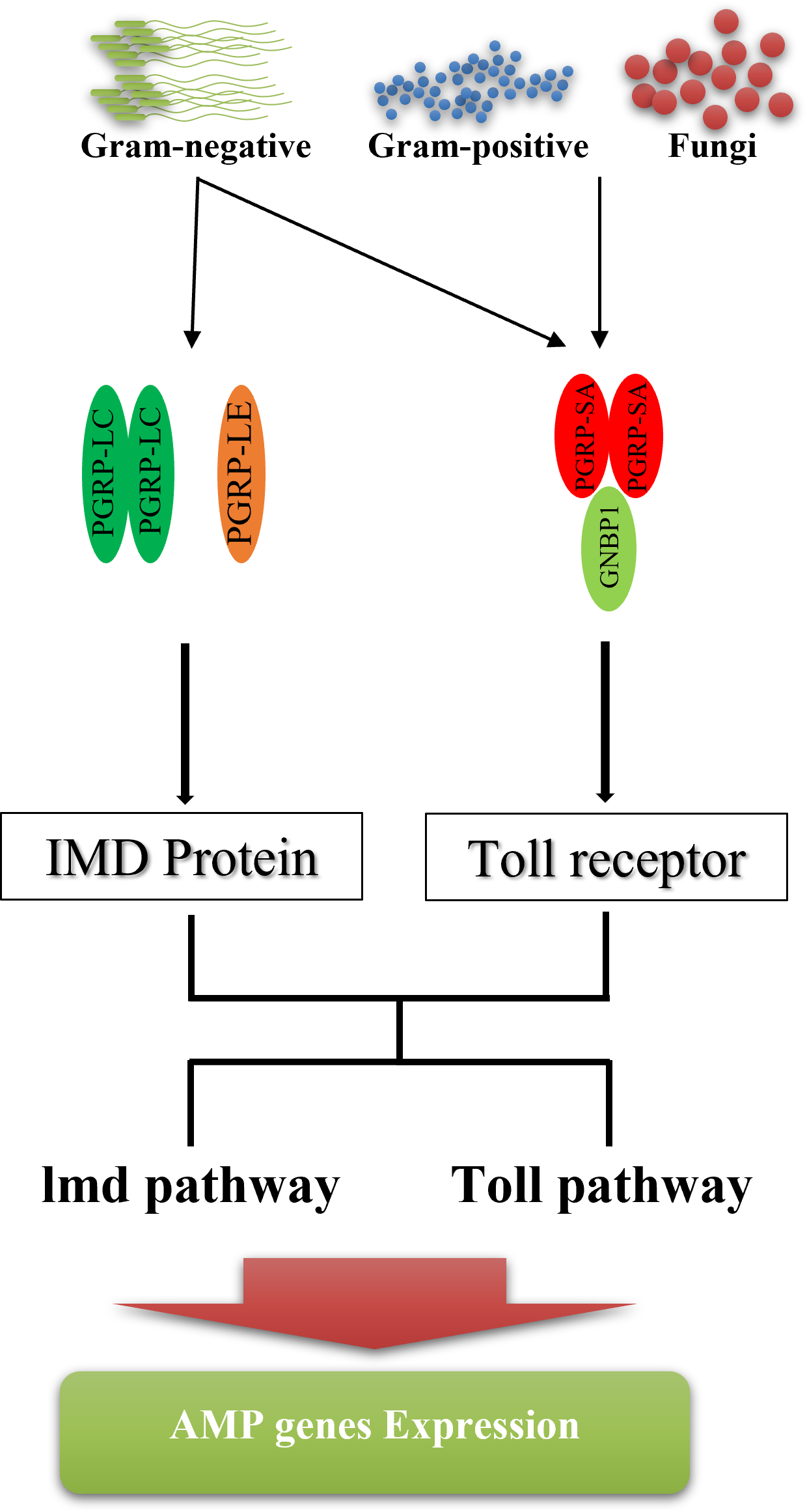

Supplement: Supplementary file 1 [file ijms-24-06751-s001.zip › Supplementary Figure S1.tif]

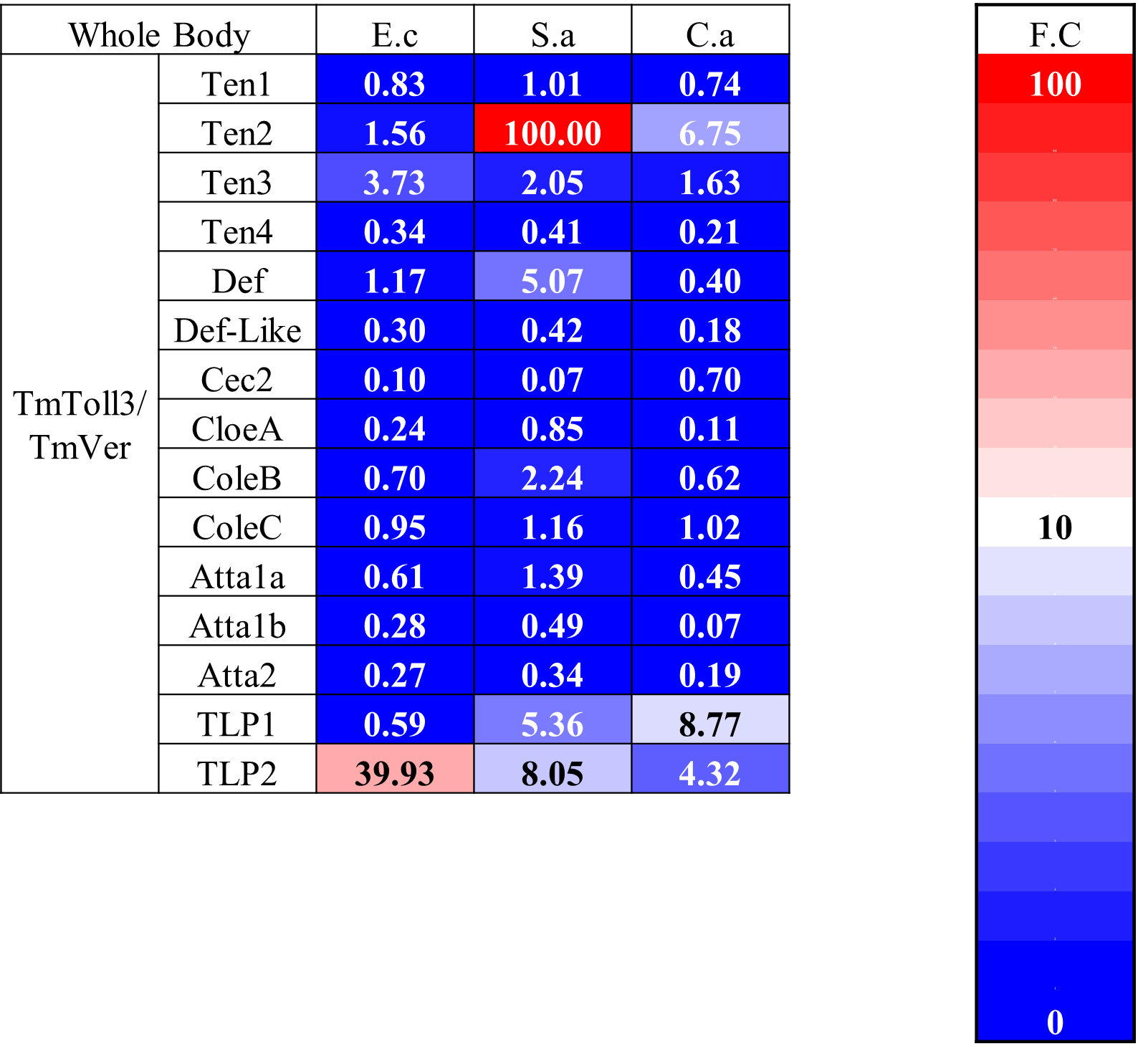

Supplement: Supplementary file 1 [file ijms-24-06751-s001.zip › Supplementary Figure S2.tif]
